# Effects of anethum graveolens and garlic on lipid profile in hyperlipidemic patients

**DOI:** 10.1186/1476-511X-6-5

**Published:** 2007-03-01

**Authors:** Javad Kojuri, Amir R Vosoughi, Majid Akrami

**Affiliations:** 1 Department of Cardiology, Shiraz University of Medical Sciences, Shiraz, Iran

## Abstract

**Background:**

hyperlipidemia as a major risk factor of atherosclerosis is treated with different drugs. Concerning length of therapy and vast majority of side effects, herbal medication may be suitable substitute for these drugs.

**Methods:**

In this single-blind, placebo controlled study, lipid profiles of 150 hyperlipidemic patients in cardiology outpatient department of Shiraz University of Medical Sciences were checked at same conditions. They were divided into three equal groups randomly (each composing of 50 patients). They were given enteric-coated garlic powder tablet (equal to 400 mg garlic, 1 mg allicin) twice daily, anethum tablet (650 mg) twice daily, and placebo tablet. All patients were put on NCEP type Π diet and Six weeks later, lipid profiles were checked.

**Results:**

In garlic group: total cholesterol (decreased by 26.82 mg/dl, 12.1% reduction, and P-value: .000), and LDL-cholesterol (decreased by 22.18 mg/dl, 17.3% reduction, and P-value: .000) dropped. HDL-cholesterol (increased by 10.02 mg/dl, 15.7% increase, and P-value: .000) increased. Although triglyceride dropped by 13.72 mg/dl (6.3%) but this was not significant statistically (P-value: .222). In anethum group: surprisingly, triglyceride increased by 14.74 mg/dl (6.0%). Anethum could reduce total cholesterol by 0.4 % and LDL-cholesterol by 6.3% but these were not significant statistically (P-value: .828, and .210, respectively).

**Conclusion:**

Anethum has no significant effect on lipid profile, but garlic tablet has significant favorable effect on cholesterol, LDL-cholesterol, and HDL-cholesterol. Garlic may play an important role in therapy of hypercholesterolemia.

## Background

Atherosclerosis remains the major cause of death and premature disability. Hyperlipidemia is the most firmly established and best understood risk factor for atherosclerosis [1]. Nowadays, major drugs used for treatment of hyperlipidemia have several adverse effects. Herbal medications such as Garlic (Allium sativum) and Anethum graveolens are prescribed as antihyperlipidemic agents.

The medicinal uses of garlic have a long history [2]. Recent studies have validated many of its useful properties, such as:

• Cancer-preventive actions [3-5]

• Stimulation of phagocytotic function of macrophages and lymphocyte proliferation [6]

Antimicrobial effects. In vitro, allicin, the main organosulfur compound of garlic, has demonstrated activity against gram-positive and gram-negative bacteria as well as fungi, protozoa, and certain viruses [7].

• The cardiovascular-protective effects of garlic have been evaluated extensively in recent years. In animal experiments, garlic extracts have been shown to lower plasma lipid and cholesterol in rats [[8,9], and [10]], rabbits [11], chickens [12], and swine [13]. Moreover, a number of intervention studies have similarly shown that garlic significantly reduced plasma lipids, especially total cholesterol and Low Density Lipoprotein (LDL) cholesterol in humans [[14,15], and [16]]. Aside from the reported antiplatelet aggregation and antithrombotic action [17], garlic reduced blood pressure [[18,19], and [20]] and stimulated fibrinolytic activity [21,23]. It was reviewed that aged garlic extract contains antioxidant compounds and increase nitric oxide production and decreases the output of inflammatory cytokines from cultured cells. These data suggest that garlic may improve impaired endothelial function in men with coronary disease treated with aspirin and statin [22]. Two meta-analyses of randomized, placebo-controlled human studies confirmed the hypocholesterolemic effects of garlic [24,25]. The analyses further detected that the extent of the cholesterol-lowering properties of garlic differed markedly from one study to another [24,25]. It was estimated from the five randomized clinical trials that hypercholesterolemic patients treated with garlic had a mean plasma cholesterol concentration that was 9% lower than that of patients treated with placebo [25]. Silagy and Neil [25], on the other hand, concluded from the analysis of 17 human studies that plasma cholesterol concentrations of the subjects treated with garlic were 12% lower than those receiving placebo. Furthermore, the two analyses detected a wide range of decrease in mean plasma cholesterol concentrations (i.e., 6–53 mg/dl) among the studies. However, garlic supplementation has been shown not to decrease plasma cholesterol concentrations in human [[26,27], and [28]]. Although the reasons for the inconsistent observations are not readily apparent, it is worthwhile to note that garlic contains a variety of organosulfur compounds, amino acids, vitamins and minerals [2]. Some of the sulfur compounds such as allicin, ajoene, S-allylycysteine (SAC), diallyl disulfide (DADS), S-methylcysteine sulfoxide, and S-allylcysteine sulfoxide may be responsible for the therapeutic properties of garlic [9]. Animal studies have shown that garlic supplementation in the diet depressed the hepatic activities of lipogenic and cholesterogenic enzymes such as 3-hydroxy-3-methyl-glutaryl-CoA(HMG-CoA) reductase [29].

Anethum graveolens is another herbal medication with antihyperlipidemic effects. Administration of water extract of anethum graveolens leaves for 14 days can reduce triglyceride and total cholesterol levels by almost 50 % and 20 %, respectively [30].

Nowadays, there is wide spread belief among general public that garlic and anethum graveolens have beneficial effects on hyperlipidemia. This belief persuaded us to perform this investigation.

## Materials and methods

A single-blind, randomized, placebo-controlled intervention study was conducted on patients with coronary artery disease with newly diagnosed hyperlipidemia. One hundred and seventy two patients were selected from cardiology outpatient department of Shiraz University of Medical Sciences. Exclusion criteria included; having significant hepatic, renal and gastrointestinal tract disease, acute myocardial infarction, uncontrolled endocrine disease, and underlying previous therapies for hyperlipidemia. Twenty two cases were excluded. One hundred and fifty patients had total cholesterol level ≥ 200 mg/dl and/or LDL-cholesterol ≥ 100 mg/dl after 10 hours of fasting with standard enzymatic methods. Also, we checked HDL-cholesterol and triglyceride. Written informed consent was obtained from each patient before any study-specific procedure.

We randomly divided patients into three groups, each composing of 50 cases. Garlic group received enteric-coated garlic powder tablet (equal to 400 mg garlic and 1 mg allicin) twice daily. Patients of Anethum group were prescribed anethum tablet 650 mg twice daily. Placebo group were given placebo. Demographic information of different groups is in table 1. After 6 weeks, we checked total cholesterol, LDL-cholesterol, HDL-cholesterol, and triglyceride after 10 hours of fasting.

**Table 1:** Demographic characteristics of different groups

	**Garlic group**	**Anethum group**	**Placebo group**	**Total**
Numbers	50	50	50	150
Female	32	33	30	95
Male	18	17	20	55
Age (year)				
Mean	55	55.56	56.5	55.8
Minimum	39	31	39	31
Maximum	78	75	74	78

All patients were given NCEP type Π, i.e. protein about 0.6 g/kg desirable body weight per day, 55 % of total calories from carbohydrate, no more than 30 % of total energy intake be derived from dietary fat, polyunsaturated fats to <10 %, saturated fat & trans-fat should be limited to <10 % of calories, throughout the study.

Data were analyzed by paired sample *t *test and non parameter 2 related sample test using SPSS 13.0 program for windows (SPSS Inc. Chicago, Illinois). A difference was considered statistically significant when the probability value (P-value) was < 0.05.

## Results

Fortunately, all of the patients cooperated well and did not show any adverse effect of these herbal drugs.

Changes of lipid profiles after 6 weeks and results of paired *t *test are shown in table 2 and 3, respectively. It was found that changes in triglyceride, total cholesterol, LDL-cholesterol, and HDL-cholesterol were significantly different between three groups.

**Table 2:** Changes in lipid profile after 6 weeks

	**Garlic**		**Anethum**		**Placebo**	
	**Mg/dl**	**percent**	**Mg/dl**	**percent**	**Mg/dl**	**percent**
Triglyceride	↓ 13.72	↓ 6.3	↑ 14.74	↑ 6.0	↑ 4.44	↑ 2.0
T C	↓ 26.82	↓ 12.1	↓ 1.12	↓ 0.4	↑ 4.6	↑ 2.0
LDL	↓ 22.18	↓ 17.3	↓ 9.34	↓ 6.3	↑ 2.3	↑ 1.6

HDL	↑ 10.02	↑ 15.7	↓ 1.4	↓ 3.1	↓ 2.14	↓ 4.6

T C: Total Cholesterol, LDL: Low Density Lipoprotein Cholesterol, HDL: High Density Lipoprotein Cholesterol

**Table 3:** Results of paired *t *test in different groups.

	**mean**	**Standard deviation**	**95% confidence interval of the difference**		**t**	**P-value**
			**Lower**	**Upper**		
Garlic						
TG1 – TG2	13.72	78.38	-8.56	36.00	1.238	0.222
TC1 – TC2	26.82	33.61	17.27	36.37	5.642	0.000
LDL1–LDL2	22.18	31.91	13.11	31.25	4.915	0.000
HDL1–HDL2	-10.02	10.55	-13.02	-7.02	-6.715	0.000
Anethum						
TG1 – TG2	-14.74	73.59	-35.65	6.17	-1.416	0.163
TC1 – TC2	1.12	36.24	-9.18	11.42	0.219	0.828
LDL1–LDL2	9.34	51.96	-5.43	24.11	1.271	0.210
HDL1–HDL2	1.42	10.30	-1.51	4.35	0.975	0.334

Placebo						
TG1 – TG2	-4.44	30.66	-13.15	4.27	-1.024	0.311
TC1 – TC2	-4.60	21.76	-10.78	1.58	-1.495	0.141
LDL1–LDL2	-2.30	14.71	-6.48	1.88	-1.106	0.274
HDL1–HDL2	2.14	7.90	-0.10	4.38	1.916	0.061

T C: Total Cholesterol, LDL: Low Density Lipoprotein Cholesterol, HDL: High Density Lipoprotein Cholesterol, TG: Triglyceride

### Placebo

Placebo could not improve lipid profile. Triglyceride, total cholesterol, and LDL-cholesterol increased and HDL-cholesterol was reduced. These changes were not meaningful (P-value > 0.05).

### Anethum graveolens

Anethum didn't have any beneficial effect on triglyceride (6.0% increase) and HDL-cholesterol (3.1% reduction). It could reduce total cholesterol by 0.4 % and LDL-cholesterol by 6.3 %, but these reductions were not significant statistically (P-value: .828, .210, respectively).

### Garlic

At the end of the six-week intervention period, it was fond that changes in triglyceride, total cholesterol, LDL-cholesterol, and HDL-cholesterol were significantly different from other groups. The mean total cholesterol concentration dropped in the garlic group by 26.82 mg/dl (P-value: .000.) Similarly, LDL-cholesterol was reduced in this group by 22.18 mg/dl (P-value: .000). Surprisingly, HDL-cholesterol was increased by 10.02 mg/dl (P-value: .000). Although triglyceride dropped by 13.72 mg/dl but this was not meaningful (P-value: .222).

## Discussion

### Garlic

Our study suggests that garlic reduces total cholesterol & LDL-cholesterol. This is similar to Alder and Holub's study (11.5% decrease of total cholesterol, and 14.2% decrease of LDL-cholesterol) [31], Tohidi and Rahbani's trial (9.0% decrease of total cholesterol, 15.0% decrease of LDL-cholesterol)[32], study of Steiner et al (6.1% decrease of total cholesterol, 4.0% decrease of LDL-cholesterol) [18]. Also, other studies explained these benefits [24,25].

HDL-cholesterol should be investigated in more clinical trials. Stevinson et al [33] in meta-analysis of randomized clinical trials on antihyperlipidemic effect of garlic explained that a slight increase in HDL-cholesterol level in the garlic group was not significantly different from the effect of placebo. Our search could corroborate garlic effect on increasing HDL-cholesterol (P-value: .000, HDL-cholesterol increased by 15.7%)

Triglyceride dropped by prescription of garlic tablet by 6.3% (13.72 mg/dl), according to Tohidi and Rahbani's study [32], and Mader's trial [34]. But due to P-value: .222, this reduction was not significant difference from the effect of placebo.

### Anethum graveolens

Anethum could reduce total cholesterol and LDL-cholesterol (1.12 mg/dl and 9.34 mg/dl, respectively) but these differences were not significant (P-value: .828 and .210, respectively). Surprisingly, it reduced HDL-cholesterol (3.1%) and increased triglyceride level by 6.0%. Our study on anethum is opposite to that of Yazdanparast and Alavi [34].

In conclusion we found that enteric-coated garlic tablet can reduce total cholesterol, LDL-cholesterol and increase HDL-cholesterol, with no effect on triglyceride. On the other hand, anethum graveolens can not be an antihyperlipidemic agent.
